# Is a GnRH Antagonist Protocol Better in PCOS Patients? A Meta-Analysis of RCTs

**DOI:** 10.1371/journal.pone.0091796

**Published:** 2014-03-18

**Authors:** Haiyan Lin, Yu Li, Lin Li, Wenjun Wang, Dongzi Yang, Qingxue Zhang

**Affiliations:** Department of Gynecology & Obstetrics, Sun Yat-sen Memorial Hospital of Sun Yat-sen University, Guangzhou, China; Azienda Policlinico S. Orsola-Malpighi, Italy

## Abstract

**Objective:**

To review published randomized controlled trials (RCTs) evaluating the outcomes of in vitro fertilization/intra-cytoplasmic sperm injection (IVF/ICSI) utilization of gonadotropin-releasing hormone (GnRH) antagonists for ovarian stimulation in polycystic ovarian syndrome (PCOS) patients compared with classic luteal long agonist protocols.

**Design:**

A meta-analysis of prospective randomized trials published in English between 2002 and 2013.

**Patient(s) and Interventions:**

Nine RCTs examining PCOS patients undergoing IVF/ICSI including 588 women who underwent long agonist protocols and 554 women who underwent GnRH antagonist protocols.

**Main Outcome Measure(s):**

Clinical pregnancy rate (CPR), ongoing pregnancy rate (OPR) and ovarian hyperstimulation syndrome (OHSS) rate.

**Result(s):**

Nine RCTs were included in this analysis. The CPR-per-embryo transferred was similar in the two groups (relative risk (RR): 0.97, 95% confidence interval (CI): 0.85–1.10). Non-significant estimates comparing the two protocols were found for age, BMI, total dose of gonadotropin administered, number of days of stimulation and number of oocytes retrieved. After meta-analysis of 4 of the RCTs, it was concluded that a GnRH antagonist protocol is better than an agonist long protocol to reduce the rate of severe OHSS (odds ratio (OR): 1.56, 95% CI: 0.29–8.51).

**Conclusion(s):**

With respect to CPR, a GnRH antagonist protocol is similar to a GnRH agonist long protocol. However, for severe OHSS, a GnRH antagonist protocol is significantly better in PCOS patients.

## Introduction

The first reports of gonadotropin-releasing hormone (GnRH) agonists for in vitro fertilization (IVF) were published in the 1980s. The function of GnRH agonists to suppress luteinizing hormone (LH) and prevent premature LH surges allowed optimal timing of human chorionic gonadotropin (hCG) administration and ovum collection, which improved IVF outcomes with respect to pregnancy rates [1]. Since that time, GnRH agonist long protocols have been the standard and mostly commonly used protocols.

Polycystic ovarian syndrome (PCOS) is the most common endocrinopathy of women of reproductive age and is characterized by oligomenorrhea, hyperandrogenism and a cystic appearance of the ovaries. Elevated LH levels are thought to be responsible for the high androgen levels, which adversely affect the development of follicles. Theoretically, blockade of endogenous LH secretion by antagonists combined with ovulation induction could result in improved follicular development. Women with PCOS undergoing IVF are at risk for a higher rate of ovarian hyperstimulation syndrome (OHSS). Triggering ovum final maturation with hCG is an important mechanism in OHSS. To overcome this barrier, GnRH antagonist protocols that use GnRH agonist triggering emerged. Since the publication of Hesham's [2] meta-analysis of 5 randomized controlled trials (RCTs), another series of RCTs [3]–[19] has been published.

These studies all used the OHSS rate as the primary outcome measurement, but there was no consensus on the classification system for OHSS between the different study sites, which limited the validity of meta-analysis. Theoretically, a GnRH antagonist protocol could reduce the OHSS rate. In addition, perhaps it is not necessary to compare OHSS rates as the primary outcome measurement. Thus, the clinical pregnancy rate (CPR) was used as the primary outcome measurement in this analysis, and the aim of this meta-analysis was to compare IVF outcomes for GnRH agonist long protocols and GnRH antagonist protocols in women with PCOS using the highest quality and most recent of the available data.

## Materials and Methods

### Criteria for considering studies for this review

Studies that compared long agonist protocols with GnRH antagonist protocols in PCOS patients undergoing IVF were considered for this review. The PCOS diagnosis had to fulfill the Rotterdam consensus criteria (Rotterdam ESHRE/ASRM-sponsored PCOS consensus workshop group, 2004). Information regarding patients and cycle characteristics such as age, number of oocytes retrieved and pregnancy outcomes was also required.

### Search strategy to identify studies

Studies were identified by searching the electronic literature through PubMed for relevant reports published between 2002 and 2013. A search strategy was employed based on the following medical subject headings (MeSH): ‘polycystic ovary syndrome’ AND ‘fertilization in vitro’ OR ‘reproductive medicine’ OR ‘reproductive techniques, assisted’ AND ‘GnRH agonist’ OR ‘GnRH antagonist’ with the time limitation of 2002–2013. In addition, the Google Scholar database was similarly searched for studies related to this topic from 2002–2013. Finally, the bibliographies of the identified studies were hand searched. Only RCTs were included in this systematic review. The databases were electronically searched in May 2013. In addition, the references of all of the selected studies were searched manually. Only abstracts written in English were considered. Excluded studies included retrospective and uncontrolled studies, editorials, reviews and meta-analyses.

### Identification

**Figure 1:**
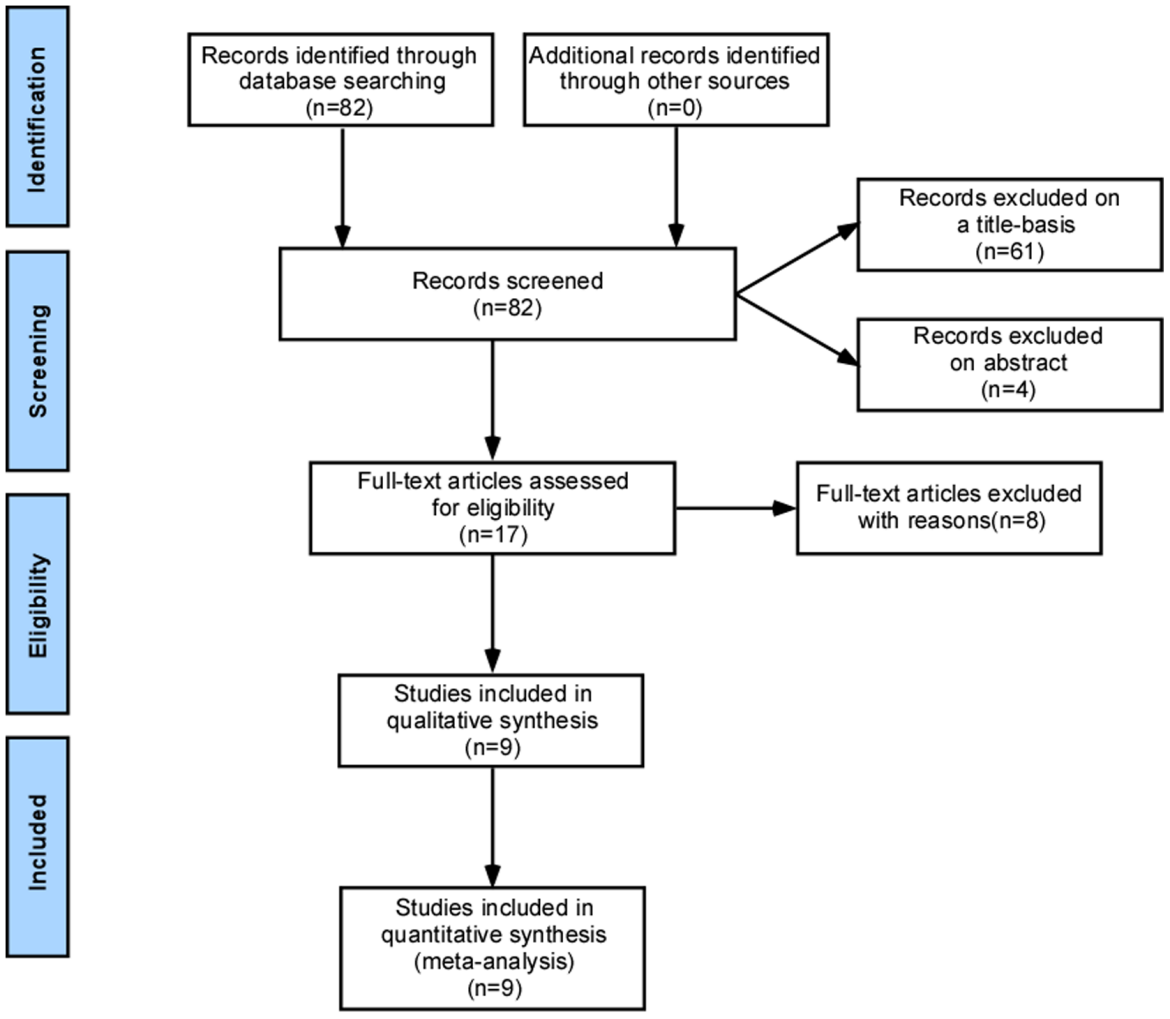
Flow Diagram.

Eighty-two potentially relevant studies were identified. Of these, 61 studies were excluded because it was clear from the title that they did not fulfill the selection criteria. Four were excluded based on the abstract. The remaining 17 studies were read in full by 2 independent researchers (Haiyan Lin and Yu Li) to evaluate whether they fulfilled the inclusion criteria. Eight of these manuscripts were excluded, and nine [4]–[8], [10], [12], [14], [17] were considered potentially appropriate for the meta-analysis (Figure 1). All of the bibliographies of the included publications were reviewed, and no additional articles were identified. The supporting PRISMA checklist are available as supporting information; see Checklist S1.

### Review methods

**Figure 2:**
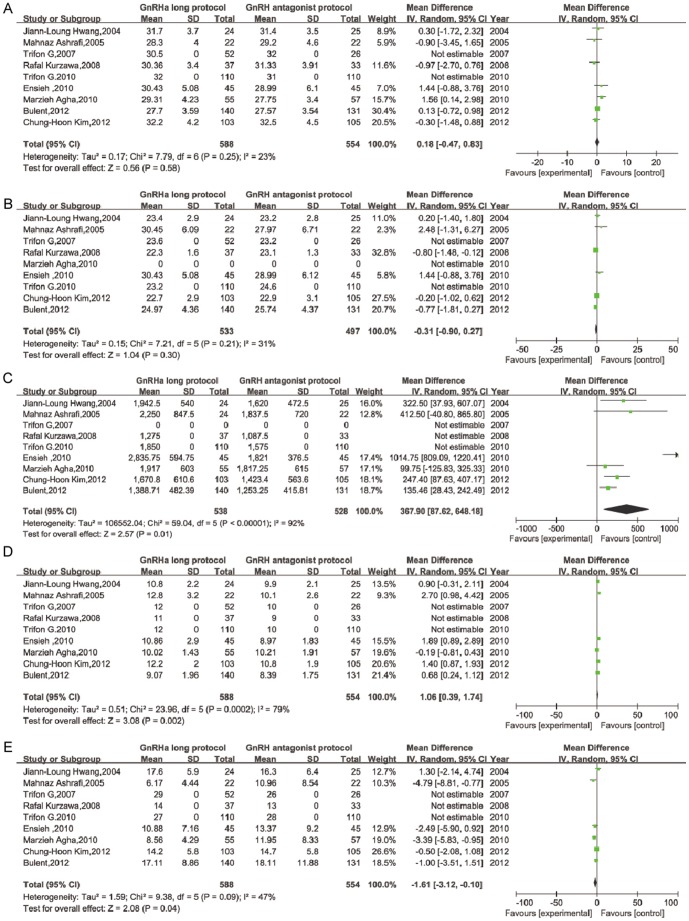
IVF/ICSI cycle outcomes of GnRH long agonist protocol versus GnRH antagonist protocol, per woman. (A)age. (B) BMI. (C) T otal dose of gonadotrophin consumed. (D) Total duration of gonadotrophin stimulation. (E) Number of oocytes retrevied.

Nine prospective RCTs were identified that addressed our research question. The study characteristics and participant features were extracted from each study. The main characteristics and level of quality of the included studies are summarized in Table 1. All nine studies had parallel designs. The primary endpoint was the number of clinical pregnancies. Secondary endpoints such as age, BMI, dose of gonadotropin, number of days of stimulation, number of oocytes retrieved and number of patients with OHSS are summarized in Figures 2 and 5. Variations in the duration and initiation time for the GnRH antagonists were observed in the different GnRH antagonist protocols.

**Table 1:** Characteristics of the studies included the meta analysis of long agonist versus antagonist protocols in polycystic ovary syndrome.

Author and Year	study size(n1/n2)	RCT	Inclusion criteria	Long agonist protocol		Antagonist protocol	
				OC pretreatment	Protocol	OC pretreatment	Protocol
**Hwang et al., 2004**	24/25	yes	at least one of the two hormonal abnormalities. The exclusion criteria: included patients older than 38 years or with serum FSH levels>.12 mIU/ml.	no	Buserelin acetate 500 mg/day was administrated from day 3 of induced or spontaneous menstruation. for 14 days, dosage was decreased to 250 mg/day at the beginning of HMG administration. until the day of HCG injection.	yes	Cetrorelix acetate was then initiated with a single dose of 0.25 mg administered on day 3, From day 4 to day 9 reduced to 0.125 mg/day, increased to 0.25 mg/day from day 10 until the day before HCG
**Mahnaz Ashrafi, 2005**	24/23	yes	thyroid tests and prolactin were normal. PCOS patients under 35 years old	yes	standard long GnRH analogue protocol	yes	Then GnRH antagonist (0.25 mg) was administered from 6th day after HMG initiation (LH≤5 IU/ml) to the day of HCG injection
**Trifon G.Lainas 2007**	52/26	yes	age 18–39 years, less than three previous IVF/ICSI attempts, no endometriotic cyst present as assessed by transvaginal ultrasound examination and basal hormonal levels of FSH in the early follicular phase of<10 IU/L. previous poor ovarian response were excluded.	yes	administered s.c. GnRH agonist 0.1 mg triptorelin daily started 3 days before discontinuation of the oral contraceptive.	yes	A daily s.c. dose of 0.25 mg ganirelix on Day 2 of menses (Day 1 of stimulation) that followed the discontinuation of the OCP.
**Rafal Kurzawa 2008**	37/33	yes	age≤35 years; body mass index<26 kg/m^2^; FSH<12 mIU/ml on the third day of the cycle. Exclude: ≥2 miscarriages, ≥3 unsuccessful IVF/ICSI cycles, anatomical abnormalities of the uterus on laparoscopy or hysteroscopy and existence of ovarian cysts.	yes	on days 16–18 of the preceding cycle, after transvaginal ultrasonographic screening of ovaries, an intramuscular injection of GnRH agonist triptorelin	yes	cetrorelix 0.25 mg was administered daily subcutaneous injections when at least two ovarian follicles reached 14 mm in diameter
**Marzieh Agha Hosseini. 2010**	55/57	yes	less than 35 years of age with a normal BMI of <27 kg/m2), normal prolactin and thyroid hormone levels	yes	GnRH agonist buserelin 500 mg subcutaneously was started on day 21 of the previous cycle.	yes	When a follicllar diameter of 14 mm was obtained, cetrorelix 0.25 mg/day was injected subcutaneously for 3 subsequent days.
**Trifon G. Lainas, 2010**	110/110	yes	age 18–39 years, no endometriotic cyst present, basal hormonal levels of FSH in the early follicular phase of <10 IU/ml. Patients with known previous poor ovarian response were excluded.	yes	GnRH agonist 0.1 mg triptorelin daily, decreased on that day to 0.05 mg/day and continued until and including the day of triggering	yes	flexible GnRH antagonist protocol daily s.c administration of ganirelix 0.25 mg initiated when at least one of the following criteria were fulfilled: (i) the presence of at least one follicle measuring .14 mm; (ii) serum E2 levels .600 pg/ml; and (iii) serum LH levels >10 IU/l.
**Ensieh Shahrokh, 2010**	45/45	yes	<35 years basal FSH<10 IU/L and undergoing their first cycle of the ART. excluded :secondary infertility, previous IVF or ICSI, thyroid dysfunction, hyper-prolactinemia, uterine abnormality and solitary ovary.	yes	500 mcgr Buserelin per day commenced on day 19–20 of OCP cycle.	yes	Administration of 0.25 mg Cetrolix acetate SQ daily initiated as soon as the follicles reached a size of 12–14 mm and continued every morning after wards, including the day of trigger.
**Chung-Hoon Kim, 2012**	105/106	yes	aged 25 to 39 years were randomized	yes	GnRH agonist, triptorelin at a dose of 0.1 mg/day was initiated from day 18 of OC pretreatment cycle. ovarian stimulation was started and the dose of triptorelin was reduced to 0.05 mg daily and continues up to day of rhCG administration.	yes	GnRH antagonist, 0.125 mg/day was administered in the morning of stimulation day 1 and 2. When the mean diameter of lead follicle reached 13 mm, cetrorelix at a dose of 0.25 mg/day was started again and continued daily up to the day of recombinant hCG
**Bulent, 2012**	150/150	yes	PCOS (age 23∼35 y) were recruited. All selected couples were in their first IVF/ICSI cycles. Women with PCOS, BMI 20∼30 kg/m^2^ were included .excluded women with PCOS whose ovaries did not appear polycystic.	yes	ovarian downregulation was initiated with daily leuprolide acetate 1 mg beginning on day 21 of the preceding menstruation. After ovarian suppression was achieved, the dose was reduced to 0.5 mg until the day of hCG.	yes	the fixed GnRH antagonist protocol daily s.c administration of ganirelix 0.25 mg was initiated on day 6 of gonadotropin until hCG administration stimulation.

### Statistical analysis

The meta-analysis was conducted using Review Manager software (RevMan) (Version 5.2, The Nordic Cochrane Centre, The Cochrane Collaboration, 2012) to calculate odds ratios (ORs), 95% confidence intervals (CIs) and heterogeneity using the Mantel-Haenszel method, assuming random effects. Statistical pooling was performed for the following outcome parameters: age, BMI, dose of gonadotropin administered, duration of stimulation, number of oocytes retrieved, number of clinical pregnancies and rate of severe OHSS. Considering an I^2^>50% as evidence of substantial heterogeneity, a random-effect model was used for the analysis where appropriate.

## Results

In total, 588 women were randomized to treatment with a GnRH agonist long protocol, and 554 women were randomized to treatment with a GnRH antagonist protocol. Seven studies compared the mean age and BMI between the two protocols. However, two studies analyzed the medians only. Pooling the results of these nine RCTs showed no significant difference between patients treated with the long agonist protocol compared with the antagonist protocol in age (weighted mean difference (WMD): 0.18, 95% CI: −0.47–0.83) and BMI (WMD: −0.31, 95% CI: −0.90–0.27). No significant statistical heterogeneity was detected (I^2^: 23% and 31%, respectively; Figure 2).

Two studies showed the median gonadotropin dose administered. One study did not supply the BMI for comparison. The remaining 6 studies were pooled for meta-analysis. A significant difference was observed in the amount of gonadotropin administered in PCOS patients between the two protocols (WMD: 367.90, 95% CI: 87.62–648.18) in the random-effect model. Significant heterogeneity was detected between studies (I^2^: 92%) because some studies used measurements such as ampoules of gonadotropin such as human menopausal gonadotropin (hMG), while others reported the total number of units of gonadotropin.

Three studies reported data on the median duration of gonadotropin stimulation, and these data were not used in the meta-analysis calculations. The mean duration of stimulation was not significantly between the two groups. The WMD was 1.06 days (95% CI: 0.39–1.74). Significant statistical heterogeneity was detected between studies (I^2^: 79%; Figure 2).

Significantly more oocytes per retrieval cycle were obtained in patients treated with GnRH agonists compared with antagonists (WMD: −1.61, 95% CI: −3.12–−0.10). No significant heterogeneity was detected between studies.

**Figure 3:**
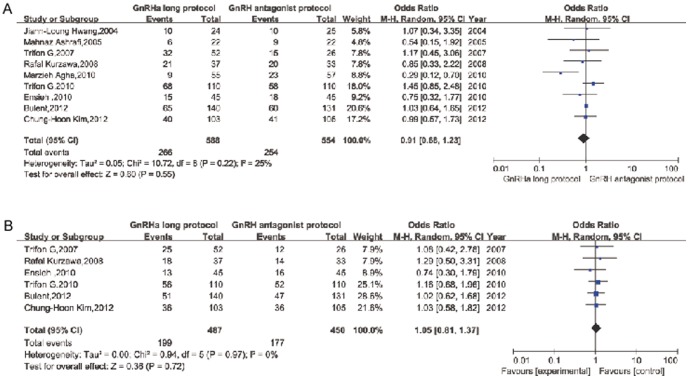
Forest plot of pregnancy and miscarriage rates for GnRH long agonist protocol versus GnRH antagonist protocol. (A) Clinical pregnancy rate. (B) Ongoing pregnancy rate.

**Figure 4:**
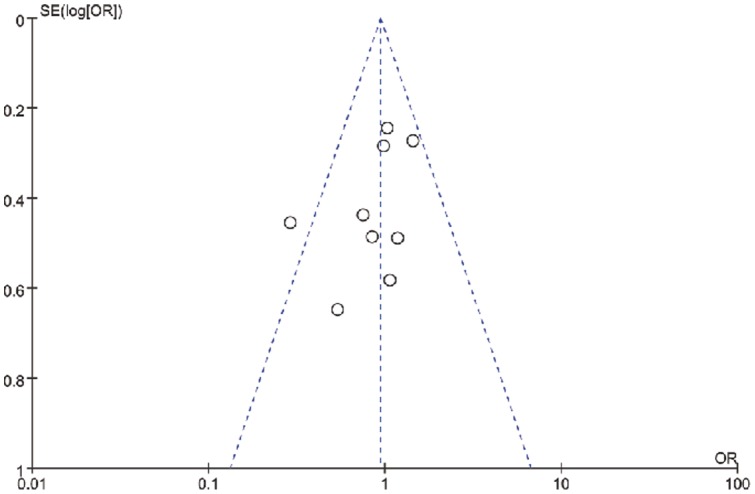
Funnel plot of the effect of GnRH long agonist protocol versus GnRH antagonist protocol on clinical pregnancy rate per embryo transfer in full analysis using Random-effects model.

The CPR per embryo transfer was reported in all nine studies. Pooling the results of all nine RCTs showed no significant difference between patients treated with the long agonist protocol compared with the antagonist protocol (OR: 0.91, 95% CI: 0.68–1.23;). No significant statistical heterogeneity was detected (I^2^: 25%; Figures 3 and 4). Six studies also reported the ongoing pregnancy rate (OPR). Pooling the results of these studies showed no significant difference between patients treated with the long agonist protocol compared with the antagonist protocol (OR: 1.05, 95% CI: 0.81–1.37); two studies reported the exact live birth rate (LBR) or delivery rate.

Data regarding fertilization rates and implantation rates were reported in different statistical measures without standard errors but with significant heterogeneity and therefore could not be pooled. All nine RCTs reported the fertilization rate, but some studies expressed it as a percentage (low and maximum interval), while others reported the median rate. Only 2 RCTs expressed the result as the mean +/− standard error. Four RCTs reported the implantation rate, while 2 RCTs reported the rate as median (range).

**Figure 5:**
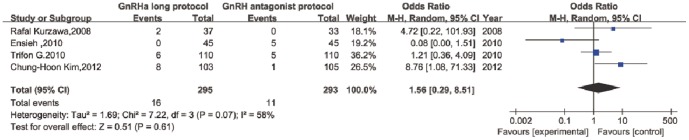
Severe OHSS rate of GnRH long agonist protocol versus GnRH antagonist protocol per woman.

In total, 8 studies recorded the rates of moderate and severe OHSS. One study simply compared the number of patients at high risk for OHSS. No cases of OHSS occurred in one study. The data on the occurrence of severe OHSS were pooled from 4 studies (OR: 1.56, 95% CI: 0.29–8.51; Figure 5).

## Discussion

After prospective randomized phase III studies comparing GnRH antagonist protocols with GnRH agonist long protocols were published in 2000–2001 [20], GnRH antagonist protocols came to be used more frequently in clinics in a wide range of patients, including patients with normal ovarian responses as well as patients with poor or high responses; this last group includes patients with PCOS.

This systematic review and meta-analysis compares two of the most commonly used ovarian stimulation protocols in women with PCOS. In GnRH antagonist protocols, a GnRH agonist can be used as an alternative to hCG as the ovulation trigger in cases at high risk for OHSS, which can be a secondary confounding factor for meta-analysis. According to the most recent systematic review [21], there was no significant difference in the incidence of severe OHSS in the antagonist group compared with the long agonist group. Thus, because it was not appropriate to perform meta-analysis of RCTs from multiple centers using the OHSS rate as a primary outcome, we used the CPR as the primary outcome in this meta-analysis.

The CPR, OPR and LBR achieved after ovarian stimulation and IVF represent very important outcome measures for any treatment protocol. There has been conflicting evidence in the literature regarding whether an antagonist protocol is as effective as a long agonist protocol with respect to CPR and OPR. Significantly lower OPRs are observed in patients on fixed 6-day GnRH antagonist cycles [22]. Furthermore, one study indicated that the stability of LH levels rather than absolute LH levels were associated with the CPR because no pregnancies occurred if the LH levels changed too markedly during GnRH antagonist administration [23]. To further test this suggestion, more large-scale RCTs comparing these regimens are required. However, the most recent Cochrane review in 2011 reported no significant difference in pregnancy rate (OR: 0.80, 95% CI: 0.57–1.11) or abortion rate (OR: 1.29, 95% CI: 0.49–3.36) between the two protocols in PCOS patients [24]. To identify a 5% difference in CPR using a β of 0.1 and an α of 0.05 with a two-tailed hypothesis test, over 1364 patients would be required in each treatment arm. Fortunately, the results of 2 large-scale studies were published in 2012.

Previous studies such the report by Xiao et al. [25] performed meta-analysis on seven studies published through 2011. We have extended that time period to include through May 2013. The present meta-analysis included nine RCTs with a total of 1142 women with PCOS and showed no significant difference between the antagonist and long agonist protocols with regard to CPR and OPR. No statistical heterogeneity was detected in the results of the included studies regarding the CPR and OPR outcomes. Thus, these results, based on a large patient population, provide further evidence that using either protocol yields a similar CPR for the treated cycle.

Meta-analysis in general has several drawbacks, such as a dependence on the quality of the reporting of the primary analysis findings and on a sufficient number of eligible studies to justify the statistical analysis. This meta-analysis has an additional disadvantage because of the different statistics regarding gonadotropin dose and duration and the lack of data on the miscarriage rates and the LBRs. Nevertheless, the findings of this meta-analysis contribute to systematizing our knowledge about outcomes of conventional IVF in women with PCOS. In our meta-analysis, we could not use the same statistics to study the total doses of gonadotropin used or the duration of stimulation due to the high statistical heterogeneity. Previous studies have all agreed that the antagonist protocol is more patient friendly and convenient [3], [12]. However, the number of scans, blood tests and clinic visits were not recorded in each study, making a full economic evaluation impossible. This convenience, added to the equal CPR compared to the agonist protocol, suggests that the antagonist protocol may be the most suitable for PCOS patients. Still, because the antagonist can be started alternatively on the first day of stimulation, on day 6 or according to the follicles' diameter, to date, no comparative studies have been performed in PCOS patients to determine which protocol is best for this population.

In conclusion, this meta-analysis did not find a statistically significant difference between the use of a GnRH antagonist protocol compared with the standard long protocol with respect to CPR and OPR in patients with PCOS undergoing IVF. Although different classifications for OHSS were used in different studies, a GnRH antagonist protocol also appears to significantly reduce the rate of severe OHSS, but further RCTs are necessary to allow more solid conclusions to be drawn. In addition, these larger RCTs should include adequate sample sizes and a standardized classification for OHSS along with economic evaluations and LBR tracking.

## Supporting Information

Checklist S1 **Supporting PRISMA checklist.**
(DOC)

## References

[1] Smitz J, Ron-El R, Tarlatzis BC (1992) The use of gonadotrophin releasing hormone agonists for in vitro fertilization and other assisted procreation techniques: Experience from three centres. Hum Reprod 7 suppl 1: 49–66. 10.1093/humrep/7.suppl_1.49 1332986

[2] Al-Inany H, Aboulghar M (2002) GnRH antagonist in assisted reproduction: a Cochrane review. Human Reproduction 17 4: 874–885. 11925376 10.1093/humrep/17.4.874

[3] Orvieto R, Meltcer S, Liberty G, Rabinson J, Anteby EY, et al (2012) Does day-3 LH/FSH ratio influence in vitro fertilization outcome in PCOS patients undergoing controlled ovarian hyperstimulation with different GnRH-analogue? Gynecol Endocrinol 28 6: 422–4. 22578028 10.3109/09513590.2011.633661

[4] Haydardedeoglu B, Kilicdag EB, Parlakgumus AH, Zeyneloglu HB (2012) IVF/ICSI outcomes of the OCP plus GnRH agonist protocol versus the OCP plus GnRH antagonist fixed protocol in women with PCOS: a randomized trial. Arch Gynecol Obstet 286 3: 763–9. 22569710 10.1007/s00404-012-2348-9

[5] Kim CH, Moon JW, Kang HJ, Ahn JW, Kim SH, et al (2012) Effectiveness of GnRH antagonist multiple dose protocol applied during early and late follicular phase compared with GnRH agonist long protocol in non-obese and obese patients with polycystic ovary syndrome undergoing IVF/ICSI. Clin Exp Reprod Med 39 1: 22–7. 22563547 10.5653/cerm.2012.39.1.22 PMC3341448

[6] Hosseini MA, Aleyasin A, Saeedi H, Mahdavi A (2010) Comparison of gonadotropin-releasing hormone agonists and antagonists in assisted reproduction cycles of polycystic ovarian syndrome patients. J Obstet Gynaecol Res 36 3: 605–10. 20598044 10.1111/j.1447-0756.2010.01247.x

[7] Tehraninejad ES, Nasiri R, Rashidi B, Haghollahi F, Ataie M (2010) Comparison of GnRH antagonist with long GnRH agonist protocol after OCP pretreatment in PCOs patients. Arch Gynecol Obstet 282 3: 319–25. 20379731 10.1007/s00404-010-1429-x

[8] Lainas TG, Sfontouris IA, Zorzovilis IZ, Petsas GK, Lainas GT, et al (2010) Flexible GnRH antagonist protocol versus GnRH agonist long protocol in patients with polycystic ovary syndrome treated for IVF: a prospective randomized controlled trial (RCT). Hum Reprod 25 3: 683–9. 20008886 10.1093/humrep/dep436

[9] Vrtacnik-Bokal E, Virant Klun I, Verdenik I (2009) Follicular oestradiol and VEGF after GnRH antagonists or GnRH agonists in women with PCOS. Reprod Biomed Online 18 1: 21–8. 19146765 10.1016/s1472-6483(10)60420-8

[10] Kurzawa R, Ciepiela P, Baczkowski T, Safranow K, Brelik P (2008) Comparison of embryological and clinical outcome in GnRH antagonist vs. GnRH agonist protocols for in vitro fertilization in PCOS non-obese patients. A prospective randomized study. J Assist Reprod Genet 25 8: 365–74. 18802744 10.1007/s10815-008-9249-7 PMC2582126

[11] Orvieto R, Meltcer S, Homburg R, Nahum R, Rabinson J, et al (2009) What is the preferred GnRH analogue for polycystic ovary syndrome patients undergoing controlled ovarian hyperstimulation for in vitro fertilization? Fertil Steril 91 4 Suppl: 1466–8. 18774558 10.1016/j.fertnstert.2008.07.1711

[12] Lainas TG, Petsas GK, Zorzovilis IZ, Iliadis GS, Lainas GT, et al (2007) Initiation of GnRH antagonist on Day 1 of stimulation as compared to the long agonist protocol in PCOS patients. A randomized controlled trial: effect on hormonal levels and follicular development. Hum Reprod 22 6: 1540–6. 17347165 10.1093/humrep/dem033

[13] Bahçeci M, Ulug U, Ben-Shlomo I, Erden HF, Akman MA (2005) Use of a GnRH antagonist in controlled ovarian hyperstimulation for assisted conception in women with polycystic ovary disease: a randomized, prospective, pilot study. J Reprod Med 50 2: 84–90. 15755044

[14] Hwang JL, Seow KM, Lin YH, Huang LW, Hsieh BC, et al (2004) Ovarian stimulation by concomitant administration of cetrorelix acetate and HMG following Diane-35 pre-treatment for patients with polycystic ovary syndrome: a prospective randomized study. Hum Reprod 19 9: 1993–2000. 15284212 10.1093/humrep/deh375

[15] Ragni G, Vegetti W, Riccaboni A, Engl B, Brigante C, et al (2005) Comparison of GnRH agonists and antagonists in assisted reproduction cycles of patients at high risk of ovarian hyperstimulation syndrome. Hum. Reprod 20 9: 2421–2425. 10.1093/humrep/dei074 15890731

[16] Tesarik J, Hazout A, Mendoza-Tesarik R, Mendoza N, Mendoza C (2006) Beneficial effect of luteal-phase GnRH agonist administration on embryo implantation after ICSI in both GnRH agonist-and antagonist-treated ovarian stimulation cycles.Hum. Reprod 21 10: 2572–2579. 10.1093/humrep/del173 16926261

[17] Ashrafi M, Moini A, Mohammadzadeh A, Ezabadi Z, Zafarani F, et al (2005) A comparative study of GnRH antagonist and GnRH agonist in PCO patients undergoing IVF/ICSI cycles. Iranian Journal of Reproductive Medicine 3 1: 14–18.

[18] Garcia-Velasco JA, Bermejo A, Ruiz F, Martinez-Salazar J, Requena A, et al (2011) Cycle cheduling with oral contraceptive pills in the GnRH antagonist protocol vs the long protocol: a randomized, controlled trial. Fertil Steril 96: 590–3. 21718992 10.1016/j.fertnstert.2011.06.022

[19] Kaur H, Krishna D, Shetty N, Krishnan S, Srinivas MS, et al (2012) A prospective study of GnRH long agonist versus flexible GnRH antagonist protocol in PCOS: Indian experience. J Hum Reprod Sci 5: 181–6. 23162357 10.4103/0974-1208.101019 PMC3493833

[20] Olivennes F, Cunha-Filho JS, Fanchin R, Bouchard P, Frydman R (2002) The use of GnRH antagonists in ovarian stimulation. Human reproductive update 8 3: 279–290. 10.1093/humupd/8.3.279 12078838

[21] Pundir J, Sunkara SK, El-Toukhy T, Khalaf Y (2012) Meta-analysis of GnRH antagonist protocols: do they reduce the risk of OHSS in PCOS? Reproductive BioMedicine Online 24: 6–22. 22133908 10.1016/j.rbmo.2011.09.017

[22] Kolibianakis EM, Zikopoulos K, Schiettecatte J, Smitz J, Tournaye H, et al (2004) Profound LH suppression after GnRH antagonist administration is associated with a significantly higher ongoing pregnancy rate in IVF. Human Reproduction 19 11: 2490–2496. 15319383 10.1093/humrep/deh471

[23] Huirne JA, van Loenen AC, Schats R, McDonnell J, Hompes PG, et al (2005) Dose-finding study of daily GnRH antagonist for the prevention of premature LH surges in IVF/ICSI patients: optimal changes in LH and progesterone for clinical pregnancy. Human Reproduction 20: 359–367. 15567880 10.1093/humrep/deh601

[24] Mancini F, Tur R, Martinez F, Coroleu B, Rodriguez I, et al (2011) Gonadotrophin-releasing hormone-antagonists vs long agonist in in-vitro fertilization patients with polycystic ovary syndrome: a meta-analysis. Gynecological Endocrinology 27 3: 150–155. 21117862 10.3109/09513590.2010.526667

[25] Xiao J, Chen S, Zhang C, Chang S (2013) Effectiveness of GnRH antagonist in the treatment of patients with polycystic ovary syndrome undergoing IVF: a systematic review and meta analysis. Gynecological Endocrinology 29 3: 187–191. 23194095 10.3109/09513590.2012.736561

